# Miscellany

**Published:** 1889-05

**Authors:** 


### MISCELLANY.

***The Johns Hopkins Hospital of Baltimore.*** —The Johns Hopkins Hospital will be formally opened May 7. The grounds of the Hospital include four entire blocks, containing about fourteen acres of ground, having a frontage on Broadway of 709 feet and extending back 856 feet. The buildings are seventeen in number and cover over four acres, and have been building about thirteen years, and are the result of the generosity of the late Johns Hopkins. The endowment is over $3,000,-000, invested in securities. Only the income has been used in the construction of the buildings. With the exception of the Laundry, Autopsy Building, Chapel and Green-house, all the buildings, which are of brick with trimmings of dark blue stone, are connected by covered corridors one story high. The top of the corridor forms an open terrace-walk on a level with the ward floors. The objects of the hospital are: The proper care of the indigent sick; the education of trained nurses and of physicians; the promotion of discovery in medical science and the promulgation of the same for the benefit of the public. The outdoor relief will tend to popularize the hospital.

The medical school will not be a part of the hospital, but the students of this department will receive their practical experience in the hospital.

### THE GENERAL PLAN.

The cottage plan adopted, with commodious arrangements, large, airy apartments given up to the sick, with an adequate supply of nurses, and of medical and surgical skill, shows a smaller average mortality than the more crowded ward system. The great objection made against placing one ward above the other is based on the increased facility for the transmission of foul air from one floor to another, and the impossibility of giving as good ventilation to the lower as to the upper floors. The plan adopted in constructing this hospital is, to have but one floor in a pavilion devoted to ward uses.

The pavilions have a basement nine feet high, raised above ground, laid with a granolithic flooring, containing only the heating and ventilating apparatus, and connected directly with a closed corridor ten feet wide, which communicates directly with all except two buildings, the dead-house and laundry. With the exception of the pay wards, none of the pavilions can be entered from the closed corridor except by first passing through the open air. All the floors on which the sick wards are situated communicate directly with a long, flat terraced roof on the top of the corridors, surrounded by railings, which serves for exercise of the patients and their general communication in fair weather, and likewise affords a free circulation of air all around the wards left unobstructed on what is termed the first floor of the system.

Each patient in the hospital is to have 100 feet of floor space, thus providing ample breathing space, and avoiding dangers common to hospitals. Not more than 400 sick persons are to be accommodated at any one time in the institution.

The common wards are rectangular in form, ninety-one feet long by thirty-two feet broad, with twelve beds arranged on either side of its length, at least one window being placed between each two. In the octagonal ward the beds are placed at exactly equal distances from each other and from the fireplaces and ventilators. A large ventilating shaft occupies the center of the room. Each of the two octagonal wards has two stories.

The isolating ward is for the treatment of patients suffering from contagious diseases. Each person is assigned a separate apartment, whose ventilation is made through an individual shaft.

Heating and ventilation are inseparably connected in making plans for hospitals. The open fireplace here has a conspicuous place in all the buildings where a room occupied by not more than two persons is to be heated.

An electrical device called a "telemeter" is to be placed in the medical director's room in the Administration Building, by which he can see at a glance the temperature of various parts of each ward in the entire hospital, and if the heat be too great or too little he can order its modulation by decreasing or increasing the amount of air in a given switch-damper that is to pass around the coils.

For the removal of the vitiated gases there are several devices employed in the same ward, including, besides the fireplace heaters, a coil of hot-water pipes near the top of the shaft, which creates a constant upward current of some little velocity from the rooms below. In the central hall of each pavilion there is in addition an enormous shaft for ventilating each entire floor. At night when the products of combustion of the gas jets are to be removed, in the morning when all the wards are to be thoroughly flushed, and in summer that this dead residuum may be removed, ventilators at the top of the room are opened. The pay wards have individual rooms, with all the features of switch-damper ventilation below and above. In the Nurses' Home is exhibited an anemometer to measure the velocity of the current upward into the ventilating shaft, which rapid egress is necessary to replenish the pure air. The attics above the wards have also the best of ventilation.

Throughout all the buildings the pipes for plumbing are exposed, so they can be examined and the source of any difficulty remedied at once. No sewer is allowed to pass directly under any building. The waste water drains directly through pipes into the Harford Run, and fecal matter flows through a large outlet, which is to be frequently flushed with large quantities of fresh water, into an immense well under the Nurses' Home, which is ventilated by a stack one hundred and twenty feet high.

No mouldings are allowed on the furniture or wood-work in any of the buildings lest shrinkage cause cracks.

The baths of the hospital are in an annex where patients who are well enough are induced to go. If unable to leave their beds, a portable bath-tub is brought to the bedside. Turkish, Russian, mercury, vapor, electric, needle, plunge, and other bath-rooms are provided for all who have them prescribed for them. Separate bath buildings are provided for males and females.

The kitchen is three stories high besides the cellar and boiler vaults. The upper story contains dining-rooms for employés and sleeping-rooms for cooks.

The entrance to the kitchen is by the closed corridor. It is on the floor below the sick wards, into which its odors cannot penetrate. All modern conveniences of steam apparatus are provided for cooking, and large hoods for carrying off the vapors to the flues. It is provided that all the garbage and waste must be at once carried out into the open air and dumped into a shute to be carried away. All the viands for the patients are carried in felt boxes, to be kept hot by this simple device, to the wards some distance off. In each ward there is, however, a nurses' kitchen, with steam tables to prepare any simple dish that may be required for an emergency or during the night.

A noticeable feature everywhere is the absence of patients' elevators, for it is believed that more comfort will be insured by raising the sick on stretchers up broad stairs with an easy rise than by subjecting them to the shock of sudden stoppages on the elevator. Fear of the rise of contaminated air through the elevator hatches also assisted in deciding on this feature.

The laundry is a one-story building and completely isolated from the rest of the group, and plays an important part in the institution. Its disagreeable vapors render its complete isolation necessary. Tubs, boilers, rotary washing machines, centrifugal wringing apparatus, and other modern devices for laundrying clothes are provided. The disinfecting chamber is in the basement of this building. The roof is flat, and is to be used for sunning the clothes.

THE DEAD-HOUSE.

The autopsy building, near the corner of Monument and Wolf streets, now used by the pathological laboratory of the University, also stands isolated for an obvious good cause. It contains on its first floor an amphitheatre, where fifty students standing, after the plan adopted in Berlin and Strasburg, can view an autopsy. There is also an air-tight morgue-room, a waiting-room for funerals, and a pathological laboratory, all on the first floor. The upper story contains rooms for histological research and for photomicrography and a small museum. The basement will probably have a small crematorium in which to consume refuse animal matter.

Three more common wards, an octagonal ward and an isolating ward, a chapel and a green-house are yet to be constructed. There will be extensive gardens connected with the hospital.

---

***A Judge's Opinion on the Use of the Title Homœopathist.***—Judge George C. Barrett, of the Supreme Court of New York, sends to the *New York Medical Times* an opinion in reply to the question: "Has a physician designating himself an 'homœopathist' and called as such to a patient, any legal or moral right to adopt other than homœopathic means in the treatment of the case?" To this Judge Barrett answers: "I have your note of the 11th inst., asking my opinion upon a question of professional ethics. In my judgment there can be but one answer to your question, and that in the negative. If I call in a medical man who designates himself a 'homœopathic physician,' it is because I do not wish to be treated allopathically, or eclectically, or otherwise than homœopathically. There is an implied understanding between myself and the homœopathist that I shall receive the treatment which, by tradition and a general consensus of opinion, means small doses of a single drug administered upon the principle of *similia similibus curantur.* If there is to be any variation from that method I have a right to be informed of it and to be given an opportunity to decide. Common honesty demands that before a confiding patient is to be drugged with quinine, iron, morphine, or other medicaments, either singly or in combination, he should be told that the 'homœopathist' has failed, and that relief can only be afforded by a change of system. An honest 'homœopath' who has not succeeded, after doing his best with the appropriate homœopathic remedies administered on homœopathic principles, should undoubtedly try anything else which he believes may save or relieve his patient. But when he reaches that point the duty of taking the patient into his confidence becomes imperative. The patient may refuse to submit to the other system or he may agree, but prefer a physician whose life has been specially devoted to practice under that other system. He may say to the 'homœopathist,' 'You have failed, but I prefer to try another gentleman of your own school, before resorting to a system that I have long since turned my back upon.' Or he may say, 'Well, if homœopathy cannot save me, I prefer to go to headquarters for allopathic treatment.' All this, gentlemen, is the logical sequence of the particular designation 'homœopathist.' "—*Medical Record*, May 4, 1889.

---

***Medical Men and their Remuneration.***—A curious case will shortly come up for trial in a law court in Saxony, the result of which will scarcely fail to be interesting to most practitioners. A medical man was in attendance upon the wife of a rich man in Saxony. The patient was exceedingly ill, and her husband was naturally extremely anxious. For the purpose, as it would seem, of stimulating the best energies of the practitioner, the husband promised, if his wife recovered, to give half his fortune to the doctor. The circumstances were propitious for the patient, and her health became established; in short, a complete cure was effected. This result having occurred to the entire satisfaction of the husband, the latter forwarded in the course of time a large sum of money in requital of the practitioner's services. The doctor, however, refused to accept the sum in question, and claimed as his reward the moiety of the fortune which had been promised him. The rich man's fortune was equal to £100,000, and although he seems to have agreed to dispose of half of this amount to the medical man in the event of the recovery of his wife, he now refuses to carry out his promise which he had given, and repudiates the claim which is being made upon him.

Thus the aid of the law is about to be sought in order to determine the point in dispute. The case for the doctor will, in all probability, entirely depend upon the legal evidence which he is able to produce of the agreement. It is scarcely to be supposed that any feeling of sentiment will be allowed to influence the result. The rash promises which are made in the presence of medical men, either by patients or their friends, is matter of almost common occurrence, under certain circumstances, while the rapid declension of that feeling which is called gratitude, after the danger is past, is proverbial, as far as the practitioner is concerned. In connection with the case above narrated, the doctor, if successful in securing £50,000, will be able to pride himself upon receiving the largest fee which has ever been paid to a medical man; but, generally speaking, it would require a sanguine person to believe that legally the rich man will be compelled to make good the promise into which he seems rashly to have entered.—*Medical Press*, April 3, 1889.

---

***Aseptic Flogging.***—A contemporary relates with apparent glee that flogging is likely to become the punishment of burglars taken with arms in their possession, and probably few people will entertain any unsurmountable objection to this additional infliction, if only *pour encourager les autres.* It is cruel, no doubt, but it is hardly less so to lodge a ball in the abdomen of the trembling householder who takes it into his head to remonstrate with the intruder. Admitting, then, that flogging may act as a deterrent, and will certainly be a punishment, we would suggest that certain precautions are desirable to prevent casualties not contemplated by the law. Unless the microbian theories are a fraud, the use of a lash steeped in the sanguineous discharge of a fellow-culprit must often be followed by the inoculation of syphilis and perhaps tuberculosis, while if the previous whipping be less recent, erysipelas, tetanus, and a host of septic complications await the victim of judicial punishment. We would suggest, therefore, that since society is "cruel only to be kind," the proper course would be to enforce the observance of antiseptic precautions by sterilizing the lash by immersion in some disinfectant solution, the formulæ for which are legion.—*Exchange.*

---

***New York Cancer Hospital.***—The Fourth Annual Report of this hospital, for the year 1888, has just been issued, and makes a very favorable showing. It says: "The experience of the past year has demonstrated still more clearly the marked advantages in hospital construction to which reference was made in the last Annual Report. The circular form of the wards built in towers secures ample light, lessens the work of the nurses, and gives an air of cheerfulness not attainable by any other arrangement, while the perfect ventilation assures a degree of freedom from unpleasant odors that could hardly be expected in such an institution. These special features, together with the architectural beauty of the building, and the careful attention to every detail that can promote the comfort and the recovery of patients, were highly commended by several of the foreign delegates to the Medical Congress at Washington last September—among others by Professor von Esmarch of Germany and Sir Spencer Wells of London."

---

***The Trained Nurse,*** edited and published in Buffalo, N. Y., has entered upon its second year. It is "consecrated to those who minister to the sick and suffering in hospital and home," and serves a good purpose in furtherance of the plan of properly educating nurses, and securing just recognition for those thus educated. The cause, and the journal devoted to the advancement of that cause, merit liberal support from the public that derives the most of the benefits resulting from their good work. It is the only journal of its kind in this country. The April number contains articles on "The Relation of Hospitals to Medical Education," "Insanity, its Causes and Cure," "Articles for the Mother's Use," "Health in our Homes," "Asepsis for the Nurse," besides considerable other editorial and original matter. The monthly Hospital Supplement contains the latest hospital news from all parts of the world.

---

***Parkes Triennial Prize.***—The prize of one hundred pounds and a gold medal for the best essay "On the Etiology and Prevention of Yellow Fever" has been awarded to Surgeon R. H. Firth, F. R. C. S. Eng., Medical Staff, now doing duty at the Station Hospital, Jullundur, in the Punjab. The subject for the next prize is "The Influence of Soil as a Factor in the Production of Disease, especially in Hot Climates," and the essays must be sent in on or before the 31st day of December, 1891. The competition is open to all medical officers of the Army, Navy, and Indian Services of executive rank on full pay, with the exception of the Assistant Professors of the Army Medical School during their term of office.

---

***Lady Medical Service in India.***—Last week the foundation stone of a new Hospital for Women was laid by Lady Lansdowne at Lucknow. The Viceroy, who was present, expressed his strong sympathy with the movement for providing medical aid for women throughout the North-West of India. It is reported that a native gentleman of Bombay has contributed 10,000 Rs. towards the cost of founding a female medical dispensary in connection with the Grant Hospital. The construction of a laboratory is being proceeded with at the latter city, destined to facilitate scientific and medical investigations and researches.

***Cremation in Berlin.***—The promoters of cremation in Berlin recently sought permission from the police authorities to cremate all those persons who during life had expressed a wish to be disposed of by fire after death. An order has been made refusing to grant the request.

***At a meeting of the Sheffield, England, Medico-Chirurgical Society,*** Mr. T. Morton showed a shawl pin 2¼ inches long which a patient, a woman, had swallowed and passed per anum at the laspe of seventeen days.

***American Medical Association.***—The Fortieth Annual Meeting of the Association will be held in Newport, Rhode Island, June 25, 1889. The officers for the year 1888-1889, are:

*President*—W. W. Dawson, M.D., Ohio. *Vice-Presidents*—W. L. Schenk, M.D., of Kansas; Frank Woodbury, M.D., of Pennsylvania; H. O. Walker, M.D., of Michigan; J. W. Bailey, M.D., of Georgia. *Treasurer*—Richard J. Dunglison, M.D., Pennsylvania. *Permanent Secretary*—Wm. B. Atkinson, M.D., Pennsylvania. *Librarian*—C. H. A. Kleinschmidt, M.D., Washington, D. C.

The Trustees of the Journal: J. M. Toner, M.D., Washington, D. C., President; John H. Hollister, M.D., Illinois, Secretary and Treasurer, E. M. Moore, M.D., New York; P. O. Hooper, M.D., Arkansas; L. S. McMurtry, M.D., Kentucky; Alonzo Garcelon, M.D., Maine; Leartus Connor, M.D., Michigan; E. O. Shakespeare, M.D., Pennsylvania; Wm. T. Briggs, M.D., Tennessee.

The Judical Council: N. S. Davis, of Illinois; H. Brown of Kentucky; Wm. Brodie, of Michigan; D. J. Roberts, of Tennessee; R. C. Moore, of Nebraska; T. A. Foster, of Maine; Jas. A. Gray, of Georgia; J. H. Murphy, of Minnesota; Jos. M. Toner, of District of Columbia; J. K. Bartlett, of Wisconsin; A. B. Sloan, of Missouri; X. C. Scott, of Ohio; B. McClure, of Iowa; W. A. Phillips, of Kansas; A. M. Pollock, of Tennessee; W. C. Van-Bibber, of Indiana; Chas. S. Wood, of New York; J. McF. Gaston, of Georgia; W. H. O. Taylor, of New Jersey; Geo. L. Porter, of Connecticut; J. F. Hibberd, of Indiana.

THE OFFICERS OF THE SECTIONS.

*Practice of Medicine, etc.*—F. C. Shattuck, Boston, Massachusetts, Chairman; G. A. Fackler, Cincinnati, Ohio, Secretary.

*Surgery and Anatomy.*—N. P. Dandridge, Cincinnati, Ohio, Chairman; W. O. Roberts, Louisville, Kentucky, Secretary.

*Obstetrics and Diseases of Women.*—W. H. Wathen, Louisville, Kentucky, Chairman; A. B. Carpenter, Cleveland, Ohio, Secretary.

*State Medicine.*—J. Berrien Lindsley, Nashville, Tennessee, Chairman; S. T. Armstrong, United States Marine Hospital, New York, Secretary.

*Ophthalmology.*—Geo. E. Frothingham, Ann Arbor, Michigan, Chairman; G. C. Savage, Nashville, Tennessee, Secretary.

*Laryngology and Otology.*—W. H. Daly, Pittsburgh, Pennsylvania, Chairman; E. Fletcher Ingals, Chicago, Illinois, Secreary.

*Diseases of Children.*—J. A. Larrabee, Louisville, Kentucky, Chairman; C. J. Jennings, Detroit, Michigan, Secretary.

*Medical Jurisprudence.*—J. G. Kiernan, Chicago, Illinois, Chairman; T. C. Evans, Baltimore, Maryland, Secretary.

*Dermatology and Syphilography.*—L. Duncan Bulkley, New York, Chairman; W. T. Corlett, Cleveland, Ohio, Secretary.

*Oral and Dental Surgery.*—F. H. Rehwinkle, Chillicothe, Ohio, Chairman; E. S. Talbot, Chicago, Illinois, Secretary.

Chairman Committee of Arrangements, R. R. Storer, M.D., Newport, Rhode Island.

---

***Canadian Medical Association.***—The General Secretary of the Canadian Medical Association, James Bell, M.D., of Montreal, has issued a circular announcing that special rates have been arranged for those who wish to attend the twenty-second annual meeting of the Canadian Medical Association, which will be held at Banff, N. W. T., on the 12th, 13th and 14th of August next.

The Canadian Pacific Railway Company has agreed to carry members and delegates with their wives or members of their families at the following rates: From points in Ontario or Quebec, to Banff and return at $95.00 each, including a double berth in sleeping car for each person, and meals in the dining cars on the way West from Montreal or Toronto and back, and four days living at the Banff Hotel.

The passage tickets will be made good from and to any points on the Canadian Pacific Railway, in either Ontario or Quebec, to Montreal or Toronto, but berths and meals will begin at these two places only.

Owing to the provisions of the Interstate Commerce law, it will be impossible to get reduced rates from points in the United States, with the exception of St. Paul, Minn., from which the following rate is offered: $60.00 to Banff and return, including meals and sleeping car accommodation *between Winnipeg and Banff only.* Delegates from the United States are therefore requested to make their own arrangements between their homes and Montreal, Toronto, St. Thomas or other points on the Canadian Pacific Railway.

In addition to the members of the Canadian Medical Association to whom that circular is specially addressed, a cordial invitation is extended to all members of the regular profession in good standing in the Dominion of Canada, the United States and Great Britain, to whom the necessary certificates will be sent on application to the Secretary.

Members and delegates are requested to notify the Secretary of the points on the Canadian Pacific Railway from which they intend to start at a sufficiently early date to enable the railway company to forward special tickets to the aforesaid points.

It will also be necessary to present a certificate from the General or Provincial Secretary to enable members or delegates to secure the above mentioned special tickets.

---

***Illinois State Medical Society.***—At the Thirty-ninth Annual Meeting of the Illinois State Medical Society, to be held in Jacksonville May 21, the committee appointed at the last annual meeting of the society to revise its constitution and by-laws should make a report. One of the resolutions adopted in creating that committee was "That said committee be required to mail every member of this society a printed copy of the proposed constitution and by-laws at least sixty days prior to the next annual meeting in 1889."

Unless that resolution has been complied with it would be well for the society to defer action on the report of the committee, if radical changes should be recommended, until the society has had sufficient opportunity to carefully examine the proposed changes.

---

***International Congress of Physiologists.***—A Congress of Physiologists will be held at Bâle, Switzerland, beginning on Tuesday, September 10th, 1889, to discuss physiological questions and such points of anatomy, histology, physics, chemistry, experimental pathology, and pharmacology as bear directly on physiology. It is stated that it is not intended to publish any record of the proceedings of the meeting.

